# Dual-career through the elite university student-athletes’ lenses: The international FISU-EAS survey

**DOI:** 10.1371/journal.pone.0223278

**Published:** 2019-10-02

**Authors:** Giancarlo Condello, Laura Capranica, Mojca Doupona, Kinga Varga, Verena Burk

**Affiliations:** 1 Graduate Institute of Sports Training, Institute of Sports Sciences, University of Taipei, Taipei, Taiwan; 2 Department of Movement, Human and Health Sciences, University of Rome Foro Italico, Rome, Italy; 3 European Athlete as Student Network, Ghaxaq, Malta; 4 Faculty of Sports, Department of Sport Sociology and History, University of Ljubljana, Ljubljana, Slovenia; 5 Institute of Sports Science, University of Tübingen, Tübingen, Germany; 6 Fédération Internationale du Sport Universitaire Education Committee, Lausanne, Switzerland; University of Lleida, SPAIN

## Abstract

Athletes have the right to combine their sport and higher education careers (e.g., dual career), but differences in the recognition of the student-athlete’s status and availability of dual career programmes and services exist worldwide. The purpose of this study was to investigate the dual career phenomenon through the international student-athletes’ views. Student-athletes competing at the 2017 Summer Universiade were recruited to respond a 31-item online survey encompassing demographic characteristics (Q1-8), sport and university engagement (Q9-13), student-athletes’ knowledge and possible sources of information regarding dual career (Q14-22); and dual career support at personal, sport, and academic levels (Q23-31). Four hundred twenty-six respondents (males: 46%, females 54%), competing in 22 different sports (individual: 74%, team: 26%) from Africa (4%), America (20%), Asia (34%), Europe (39%), and Oceania (3), had experienced previous international sports events (94%). Differences among continents emerged for sport (p<0.001) and university (p = 0.039) engagement, and transfer time from home to the training venue (p = 0.030). Individual sports student-athletes showed higher sport engagement (p = 0.003) compared to team sports counterparts. Differences among university majors emerged for university engagement (p<0.001). Long absence from classes (57%), limited leisure time (50%), financial uncertainty (44%), reduction of training due to education (42%), and overload feelings (37%) emerged. The majority of the sample resulted not familiar with dual career programmes (60%) and public authorities (69%), envisaging national dual career policies at university (37%) and sport (25%) levels. Multiple relevant dual career supporters at personal, sport, and university levels were identified, mainly parents (86%) and coaches (65%). To strengthen the potential of the student-athletes of the future, a dual career network should be established among several stakeholders, for transnational cooperation and sharing of knowledge and best practices through extensive communication between policy-makers, practitioners and those having a strong supportive dual career role (e.g., parents, coaches, and university sport staff).

## Introduction

To achieve a holistic development, talented and elite athletes have the right to combine their sport and higher education careers (e.g., dual career), both relevant to empower them for their future role in society at the end of their competitive sport period [1,2]. Although athletes enrolled at university level have the opportunity to compete at multi-sport international and continental university sport events (e.g., Universiade, World University Championships, Pan-American University Championships, European EUSA Games), relevant differences exist worldwide in the requirements and eligibility criteria for dual career programmes and services, which determine unequal treatments of elite student-athletes mainly due to country-specific cultural/organizational regulations in the field of sport and education [3,4]. In particular, recognition and support of student-athletes is formalized in Australia [5], Canada [6], New Zealand [7], and the United States [8], whereas dual career paths for talented and elite African, Asian and European athletes present a multiplicity of national approaches ranging from flexible academic programmes and financial/service support to individually negotiated agreements, when possible [3,4,9–11]. Hence, student-athletes from the five continents are presented with different opportunities in pursuing a successful dual career.

The responsibility of pursuing dual career paths does not only rely exclusively on the micro level of the single athletes according to their sex, age, practiced sport, previous international athletic experience, and university level and major, but also on the dual career culture at meso (e.g., relationships with parents, peers, teachers, coaches, sport managers), macro (e.g., organization of sport clubs/federations and educational institutions), and global (e.g., international, national, regional, local governing bodies policies) dimensions, which is also influenced by several socio-cultural, media, and economic mediators [4,12,13]. Therefore, supporting elite athletes’ education during their high-level competitive years heavily depends on the relationships between multiple stakeholders, who need a well-structured cooperation and a systematic monitoring system to withstand effective dual career programmes and policies [3,4,8].

At international level, there is a fragmented knowledge on dual career, which limits the understanding of the phenomenon determining the development and success of dual career of student-athletes. Despite an extensive research on American student-athletes enrolled at university level, in Europe there is a growing concern that elite training and competition requirements increasingly challenge education and lifestyles of athletes [14] after the introduction of the term dual career in the White Paper on Sport [15] and the endorsement of the European Guidelines on Dual Careers of Athletes [16], which recommend a minimal standard of facilitators and conditions for athletes in the Member States. Thus, an upsurge of interest in the management of dual career of athletes emerged at institutional [16–19], organizational [20], and research [3,4,9,13,14,21–23] levels. In the last decade, there has been an increased number of scholars interested in dual career issues in relation to culture and socio-cultural contexts, which have been summarized in two systematic literature reviews [14,23]. Scholars were particularly interested in athlete’s identity [24–26] and competences [27,28], the motivation of student-athletes towards academic and sport careers [25,26,29–35], and career development and transitions of athletes [1,12,36–38], the latter being the topic of a dedicated special issue of the Journal of Psychology of Sport and Exercise [22]. However, there is a need of cross-national comparative studies to uncover differences in relation to the sex, age, sport discipline, length, and level of the sporting experience, training and academic commitments, educational level, socioeconomic and contextual policy, and organizational variables [39]. In particular, there is a lack of information on how critical issues about dual career can differ worldwide and in relation to the sport typology. In fact, competition schedules are differently organized for team (i.e., spread over several months) and individual (i.e., packed periods) sports, which could affect the student-athletes’ capability to organize their dual career commitments [33].

Being specifically dedicated to athletes enrolled in tertiary education, the unique format of the Universiade global multi-sport events are central in the communication of athletic and academic image and identity of student-athletes and could provide a tremendous opportunity to monitor their awareness and perceptions of the multi-faceted dual career domains at policy, macro, and meso levels in relation to their personal academic and sport experience (e.g., micro level). In this framework, the Education Committee of the Fédération Internationale du Sport Universitaire (FISU) and the European Athlete as Student (EAS) network aimed to provide insights on the influential factors of dual career worldwide by collecting information from student-athletes competing for their respective African, American, Asian, European, and Oceanic countries at the 2017 Summer Universiade in Taipei, Taiwan.

Moving from the relationships between dual career stakeholders [4] and the dual career quality framework [3], this study considered the student-athletes central of their dual career paths and its purpose was to be a first attempt to allow a thorough interpretation of the global dual career phenomenon through the international student-athletes’ point of view. Thus, it was deemed relevant to gather relevant information on 1) socio-demographic characteristics of the elite student-athletes to frame this population; 2) the athletes’ sport and university engagement and their awareness of dual career available legislation to highlight critical aspects of dual career paths; and 3) the athletes’ personal, sport, and academic entourages to uncover the actual, direct, and regular dual career support and responsibilities. Furthermore, it has been hypothesized that sport typology and academic major could present differences in sport and academic engagement of student-athletes of different continents.

## Materials and methods

This study was carried out in accordance with the Declaration of Helsinki upon approval of the Institutional Review Board of University of Rome Foro Italico (CARD 2018/18).

### The instrument

According to the literature [3,10,12,16], conceptualization of the factors that could contribute to facilitate or hamper dual career of athletes was achieved. To reach student-athletes competing for countries of five continents (i.e., Africa, America, Asia, Europe, and Oceania), a web instrument was selected, which allowed a time and geographic flexibility in addition to multimedia and self-administration [40]. Thus, a 31-item online survey was constructed to gather information on the following major themes of dual career of athletes (S1 Appendix):

- Demographic characteristics of the sample (Q1-8), including the practiced sport, country, gender, age, university level, university major, and previous athletic experience at international events;
- Sport and university engagement (Q9-13), encompassing time devoted to sport and to transfer from home to the training site, and time devoted to university and to transfer from university to the training site, including problems in combining sport and education;
- Student-athletes’ familiarity and awareness of dual career policies, programmes, initiatives, and documents availability in their country and possible sources of information (Q14-22);
- Dual career support that athletes receive at personal, sport, and academic levels (Q23-31).

To collect data on a large sample of a heterogeneous population, close-ended questions were chosen (e.g., single or multiple response checklist type). Moreover, respondents were also allowed to elaborate further on their answers for questions related to previous athletic experience, problems in combining sport and education, possible sources of information on dual career policies, programmes and initiatives, dual career responsible bodies and monitoring systems, dual career support, and envisaged improvements at institutional and personal levels.

### Recruitment

According to the literature [41, 42], a pre-notification email providing information on the link to the FISU-EAS online survey for dual career was prepared and sent to the FISU national delegates, who were required to recruit the target population of athletes representing their country at the 2017 Summer Universiade. This recruitment procedure was deemed relevant to meet the standards of general data protection regulations and safeguarding privacy rights of personal data according to country-specific regulations. Conversely, this procedure did not allow calculation of the probability and response rates, being a not-list-based survey [40]. Participation was deemed voluntary and anonymous, and athletes were informed that they could withdraw from the study at any time without providing any reason, and incomplete response would not be considered. Informed consent was assumed with subjects’ compilation of the online survey. To increase response rates for online surveys including >20 items and demanding long time from the respondents [41], three follow-up contacts with a fifteen-day in between had been planned.

### Data reduction

Data reduction has been conducted for a series of questions. In particular, sport typology (Q1) was categorized in individual (e.g., archery, artistic gymnastics, athletics, badminton, billiards, diving, fencing, golf, judo, rhythmic gymnastics, roller sports, swimming, table tennis, taekwondo, tennis, weightlifting, and wushu) and team (e.g., baseball, basketball, football, volleyball, and water polo) sports, respectively. Continent (Q2) was categorized in Africa, America, Asia, Europe, and Oceania. Age (Q4) was categorized in 18–22, 23–27, >27 years of age. According to the European Research Council [43], University major (Q6) was categorized in Social Sciences and Humanities (e.g., Business and Administration, Communication, Environmental Sciences, Humanities, Language-Foreign Languages, Law, Political-International Sciences, and Psychological-Pedagogical-Social Sciences), Physical Sciences and Engineering (e.g., Computer Sciences, Engineering and Architecture, Mathematics, and Physics), and Life Sciences (e.g., Biomedical Sciences, Medicine, and Sport Sciences), respectively.

### Statistical analysis

Data were analyzed using the Statistical Package for the Social Science, version 24.0 (SPSS Inc., Chicago Illinois), with a level of statistical significance set at p<0.05 for all computations.

Descriptive statistics based on the data reduction was applied and frequency of occurrence (expressed in absolute values or percentages) was calculated for questions for which a single response (e.g., Q 1, 2, 3, 4, 5, 6, 7, 18, 25, 26) or multiple responses (e.g., Q 8, 13, 17, 19, 20, 21, 22, 23, 24, 27, 28, 29, 30, 31) were allowed. For Q9-12 participants were allowed to provide their own value and frequency of occurrence (expressed in absolute values or percentages) was calculated considering 3 classes of occurrence for the time engaged in sport and university (<11, 11–20, and >20 hr), and 4 classes of occurrence for the time necessary to transfer from home and university to the training venue (e.g., <30, 31–60, 61–90, and >90 min).

For inferential statistics, a double approach was used. Firstly, the Kolmogorov-Smirnov test was applied to ascertain the normality of data distribution for the data from Q9-12. In considering that data were not normally distributed, Kruskall Wallis test followed by Mann Whitney U test for pairwise comparisons were used to assess the effects of the independent variables Continent (e.g., Africa, America, Asia, Europe, and Oceania), Sport typology (e.g., individual and team sports), and University major (e.g., Social Sciences and Humanities, Physical Sciences and Engineering, and Life Sciences) on the dependent variables Sport and University engagement (express in hours per week) and Time necessary to transfer from home and university to the training venue (expressed in minutes for each way). Cohen’s effect sizes (ES)s were calculated for differences emerging in parametric statistical analysis, considering an ES ≤0.2, from 0.3 to 0.6, from 0.7 to 1.2, and >0.1.2, as trivial, small, moderate, and large, respectively [44]. Then, Chi-square tests were applied to verify the distribution of answers for Q14-16 in relation to Continent, Sport typology, and University major. Post hoc analysis was applied using the calculation of adjusted residuals with Bonferroni adjustment for p value interpretation.

## Results

### Demographics characteristics (Q1-8)

The 2017 Summer Universiade involved 7377 athletes (males: n = 4189, 57%; females: n = 3188, 43%) representing 134 countries, whereas 426 student-athletes (5.8%) participated in the present study (males: n = 198, 46%; females: n = 228, 54%). Despite higher proportions of athletes were competing from European (39%) and Asian (34%) countries with respect to Africans (4%), Americans (20%), and Oceanic (3%) ones, the respondents showed a higher proportion of European (62%) and American (16%) student-athletes with respect to their Asian (9%), Oceanic (7%), and African (6%) counterparts. Regarding the European countries of the respondents, the most represented resulted Poland (n = 44, 10%), Czech Republic (n = 32, 8%), Lithuania (n = 20, 5%), and Slovenia (n = 17, 4%). The relative picture for American countries was Argentina (n = 29, 7%), USA (n = 12, 3%), and Canada (n = 10, 2%), for Asian countries was Japan (n = 34, 8%) and Singapore (n = 4, 1%), for Oceanic countries was Australia (n = 20, 5%) and New Zealand (n = 9, 2%), and for African countries was South Africa (n = 22, 5%) and Ghana (n = 2, 1%), respectively.

**Fig 1:**
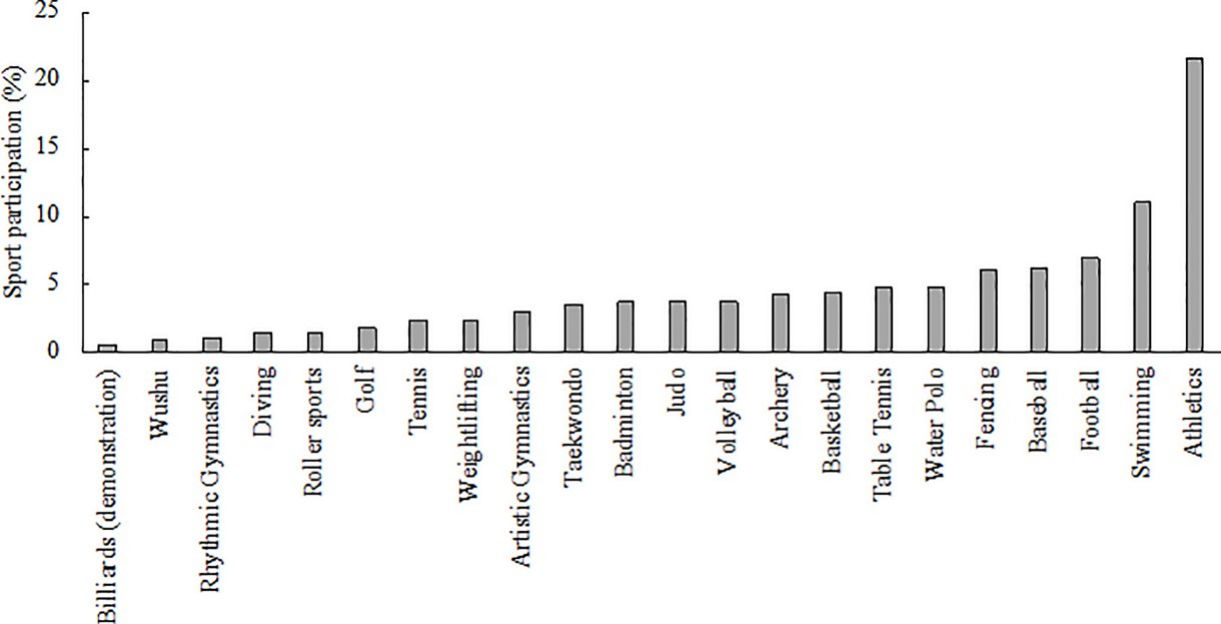
Frequency of occurrence (%) of respondents to the FISU-EAS survey in relation to their sports.

The participants in this study competed in 22 different sports (Fig 1), with a higher proportion of individual sports (74%) with respect to team (26%) ones, mirroring the different sport-specific rules to participate in the event.

Only 6% of the student-athletes were experiencing the 2017 Summer Universiade as their first international competition, whereas the remaining 94% have participated in the previous years in several international events (single and multiple participations) including world championships (n = 158), world cups (n = 111), Olympic Games (n = 35) and other international competitions (n = 339).

**Fig 2:**
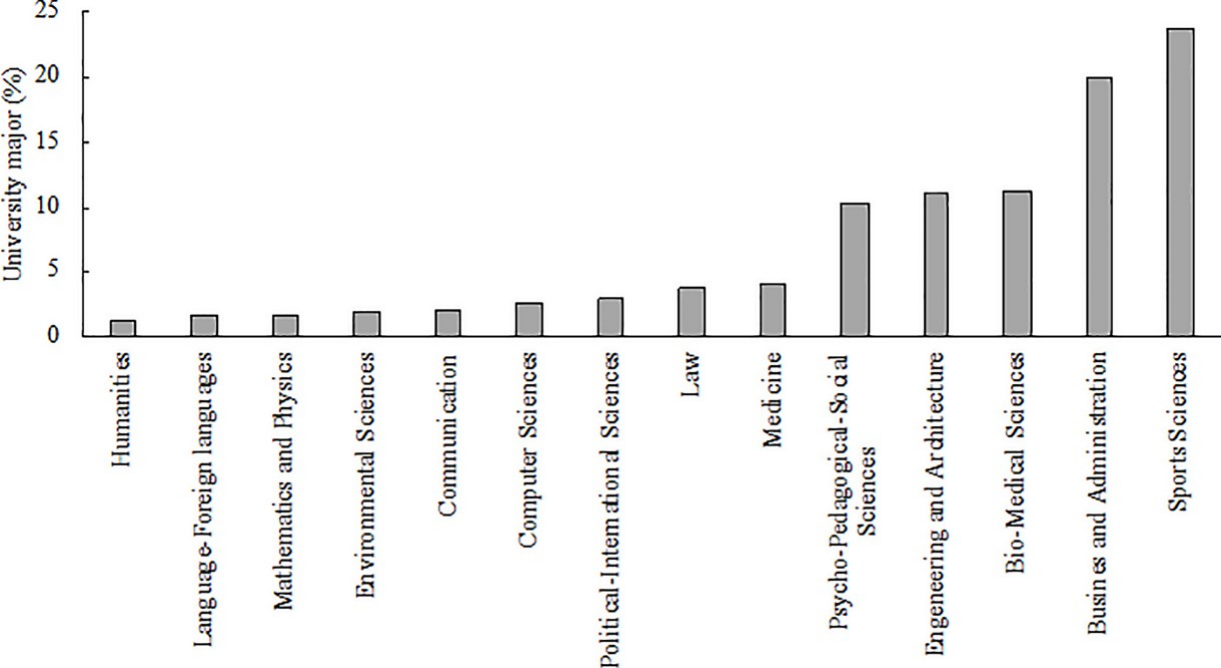
Frequency of occurrence (%) of respondents to the FISU-EAS survey in relation to their university majors.

The proportion of respondents ranging in age between 18 and 22 years was 49%, whereas that comprised in the 23–27 years and >27 years categories was 46% and 5%, respectively. Whilst the majority of participants (74%) declared to be enrolled at bachelor level, the remaining proportion was attending master’s (22%), PhD (3%), and vocational (1%) degrees. Interestingly, a wide variety of university majors emerged, with Sports Sciences being the most represented (24%) with respect to the others (Fig 2). When categorized in three University majors, the most represented was Social Sciences and Humanities (45%), followed by Life Sciences (40%) and Physical Sciences and Engineering (15%).

### Sport and university engagement (Q9-13)

**Fig 3:**
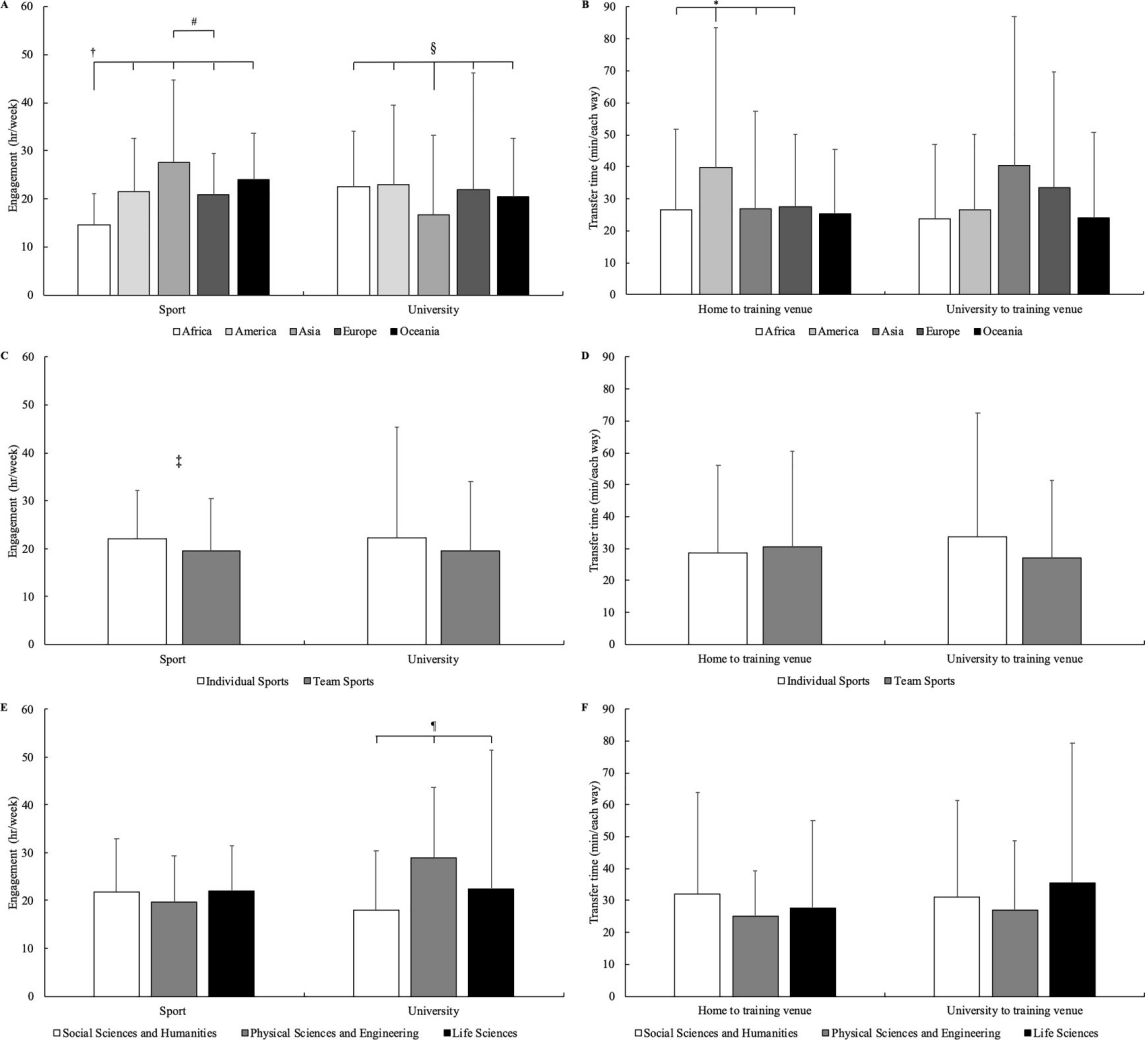
Means and standard deviations of weekly hours necessary for sport and university engagement and minutes necessary to transfer each way from home and university to the training venue in relation to continent (panel A and B), sport typology (panel C and D), and university major (panel E and F). †African student-athletes significantly different (p<0.05) from American, Asian, European, and Oceanic counterparts. #Significant difference (p = 0.017) between Asian and European student-athletes. §Asian student-athletes significantly different (p<0.05) from African, American, European, and Oceanic counterparts. *American student-athletes significantly different (p<0.05) from African, Asian, and European counterparts. ‡Significant difference (p = 0.03) between individual and team sports. ¶Significant differences (p<0.05) among the three university major categories.

No interaction was found between Continent, Sport engagement, and University engagement. Regarding Continent a main effect emerged for Sport engagement (p<0.001), University engagement (p = 0.039), and Time necessary to transfer each way from home to the training venue (p = 0.030). In relation to Sport engagement, the post hoc analysis confirmed that African student-athletes (14.7±6.5 hr·week^-1^) showed lower values with respect to their American (21.4±11 hr·week^-1^; p = 0.004; ES = 0.74), Asian (27.6±17.2 hr·week^-1^; p = 0.004, ES = 0.99), European (20.9±8.5 hr·week^-1^; p<0.001; ES = 0.82), and Oceanic (23.9±9.7 hr·week^-1^; p<0.001; ES = 1.11) counterparts. Moreover, European student-athletes resulted less engaged in Sport (p = 0.017; ES = 0.49) with respect to their Asian counterparts. In relation to University engagement, the post hoc analysis confirmed that Asian student-athletes (16.7±16.6 hr·week^-1^) showed lower values compared to their African (22.5±11.4 hr·week^-1^; p = 0.01; ES = 0.41), American (23±16.5 hr·week^-1^; p = 0.012; ES = 0.38), European (22±24.2 hr·week^-1^; p = 0.005; ES = 0.26), and Oceanic (20.5±12.1 hr·week^-1^; p = 0.045; ES = 0.26) counterparts. In relation to the Time necessary to transfer each way from home to the training venue, the post hoc analysis confirmed that American student-athletes (39.9±43.7 min each way) required significantly more transfer time compared to that of their African (26.5±25.4 min each way; p = 0.038; ES = 0.38), Asian (27±30.4 min each way; p = 0.01; ES = 0.34), and European (27.6±22.7 min each way; p = 0.033; ES = 0.35) counterparts (Fig 3A and 3B). Regarding the effect of Sport typology, no significant differences between individual and team sports were found for academic engagement and for transferring from home or university to the training venue, whereas a main effect emerged only for Sport engagement, with student-athletes from individual sports (22.1±10 hr·week^-1^) showing a higher engagement (p = 0.003; ES = 0.25) compared to their team sports (19.5±10.8 hr·week^-1^) counterparts (Fig 3C and 3D). In considering the University major, a main effect emerged for University engagement (p<0.001). The post hoc analysis confirmed differences (p<0.05; ES = 0.20–0.82) among the Social Sciences and Humanities (18±12.4 hr·week^-1^), Physical Sciences and Engineering (29±14.5 hr·week^-1^), and Life Sciences (22.5±29 hr·week^-1^) categories (Fig 3E and 3F).

Only 14% of the entire sample declared a Sport engagement <11 hr·week^-1^, whereas 44% and 42% reported to be engaged 11–20 hr·week^-1^ and >20 hr·week^-1^, respectively. Whilst 27% of student-athletes declared a University engagement of <11 hr·week^-1^, 34% and 39% reported an engagement of 11–20 hr·week^-1^ and >20 hr·week^-1^, respectively. Furthermore, the majority of respondents reported to need <31 min to transfer each way from home (75%) or university (67%) to the training venue to attend a single training session.

Only a Lithuanian male doctoral student-athlete competing in athletics declared to have no problem in combining sport and studies, whereas the majority of the sample reported multiple problems, with the highest prevalence of long absence from university classes (57%) and limited leisure time (50%). In general, student-athletes equally suffered from missing university evaluations (43%) and classes (42%) due to sport, as well as from reducing their training due to education (42%), with 37% of the respondents feeling overloaded by their dual career, probably due to a limited possibility to prolong their academic career (34%). Another relevant aspect pertains the financial uncertainty, which was declared by 44% of the sample. Among the open responses (n = 13), tiredness and lack of sleep (n = 4), problems with long distance learning (n = 2), no support from the university (n = 4), limited time for research/internships/work (n = 2), and difficulty to stay focused on both sport and academic paths (n = 1) have been reported.

### Student-athletes’ familiarity and awareness of dual career policies, programmes, initiatives, and documents availability (Q14-22)

The majority of respondents declared to be not familiar with policies, programmes or measures that facilitate the combination of elite sports and studies (60%), to be not aware of dual career policies or initiatives (75%), and of the availability of dual career policy documents (84%) (Table 1). In general, internet was considered a possible source of information on dual career (15%), even though some student-athletes declared that also multiple (4%) or other (3%) means of information could be achievable. Overall, student-athletes envisaged national dual career policies at university (37%), sport (25%), and national (27%) levels, highlighting main responsibility at multiple (31%), sport (24%), and educational (16%) domains. In particular, multiple domains (44%) and universities/schools (23%) have been identified as relevant in regulating the combination of elite sports and studies. However, the majority of the sample (69%) resulted not aware of public authorities active in dual career policies, with only 18% of the sample attributing this responsibility at national level. Similarly, the student-athletes reported to ignore the criteria for dual career monitoring (61%), whereas 18% attributed them to their sporting and academic achievements, 9% to sporting achievements only, and 11% declared that no evaluation system is in place.

**Table 1:** Frequency of occurrence (n, %) of student-athletes declaring to be (e.g., yes) or to be not (e.g., no) familiar with policies, programmes or measures that facilitate the combination of elite sport and studies, to be (e.g., yes) or to be not (e.g., no and do not know) aware of dual career policies and initiatives, and to be (e.g., yes) or to be not (e.g., no and do not know) aware of the availability of policy documents in the field of dual career in relation to five continents.

	Africa	America	Asia	Europe	Oceania
**Familiarity with policies, programmes or measures**					
Yes n (%)	20 (77)	52 (76)	36 (92)	137 (52)	10 (43)
No n (%)	6 (23)	16 (24)	3 (8)	127 (48)	19 (66)
**Total n (%)**	**26 (100)**	**68 (100)**	**39 (100)**	**264 (100)**	**29 (100)**
**Awareness of policies and initiatives**					
Yes n (%)	1 (4)	15 (22)	3 (8)	78 (30)	8 (28)
No n (%)	5 (19)	14 (21)	1 (3)	50 (19)	3 (10)
Do not know n (%)	20 (77)	39 (57)	35 (90)	136 (52)	18 (62)
**Total**	**26 (100)**	**68 (100)**	**39 (100)**	**264 (100)**	**29 (100)**
**Awareness of policy documents**					
Yes n (%)	2 (8)	5 (7)	3 (7)	52 (20)	4 (14)
No n (%)	2 (7)	11 (16)	1 (3)	34 (13)	1 (3)
Do not know n (%)	22 (85)	52 (76)	35 (90)	178 (67)	24 (83)
**Total**	**26 (100)**	**68 (100)**	**39 (100)**	**264 (100)**	**29 (100)**

In relation to Continent, the frequency of occurrence showed significant differences regarding the familiarity with policies, programmes or measures that facilitate the combination of elite sport and studies (p<0.001), the awareness of the policies or initiatives on dual career (p = 0.001), and the awareness of the availability of policy documents in the field of dual career (p = 0.026). With the exception of Africa, the post hoc analysis maintained the difference for the other continents (p<0.005). Regarding the awareness of dual career policies or initiatives, the post hoc analysis demonstrated that only the “Do not know” responses of Asian and European student-athletes significantly contributed (p<0.001) to the Chi-square test value. In relation to the awareness of the availability of policy documents, the post hoc analysis showed that only the “Do not know” responses of European athletes contributed to the significant (p = 0.0014) Chi-square value. Conversely, the post hoc analysis did not maintain the main effect (p = 0.042) for Sport typology on the awareness of dual career policies or initiatives.

### Dual career support at personal, sport, and academic levels (Q23-31)

In general, the student-athletes perceived themselves to be among the few ones engaging in elite sport and higher education, indicating the <20%, 21–40% and 41–60% frequency of occurrence categories as 29%, 19%, and 20%, respectively. Furthermore, half of the sample reported no knowledge of the actual number of student-athletes who benefit from dual career support in the respective countries, with highest guesses related to the <20% and 21–40% frequency categories. Almost half of respondents (46%) declared to receive some support at multiple levels, most frequently related to educational flexibility, financial support, and availability of sport facilities at (or close to) the university. Conversely, a limited tutoring at university and sport levels has been reported. Specific to sport and academic levels, student-athletes declared to receive multiple support (sport = 66%; academic = 51%), whereas around 10% reported no support at all. At sport level, support was more frequently related to athletic performance (e.g., coaching, medical, physiotherapy, and nutritional aspects, and sport facilities), than that related to education (e.g., rooms to study, career counselling, and tutor for dual career). At university level, sport facilities, flexible class attendance and exam sessions, and individualized study plans were reported as the most frequent support, with limited dual career tutoring, encouraged employability, and career counselling. In general, student-athletes envisaged possible improvements at multiple levels (71%), especially related to financial support, educational flexibility, and sport facilities at (or close to) the university.

In general, the respondents identified multiple relevant dual career supporters at personal, sport, and university levels (Table 2), with the highest frequencies of occurrence for parents (86%), coaches (65%), university sport staff and sport teammates (46%), friends (44%), professors (36%), and sisters and brothers (36%). Although with limited percentages (range: 2.3–0.5%), parents were also reported as dual career supporters at sport and academic levels, as well as city government and National Olympic Committee at personal level, university at sport level and sport federation at academic level, probably indicating some cases where integration between dual career dimensions occurs. Finally, a lack of support emerged most frequently at the sport (16%) and academic (15%) levels, whereas it was limited at the personal one (3%).

**Table 2:** Frequency of Occurrence (%) of dual career supporters at personal, sport, and academic entourage levels calculated in relation to the number of respondents (multiple responses were allowed).

Personal Entourage		Sport Entourage		Academic Entourage	
Supporter	%	Supporter	%	Supporter	%
Parents	85.5	Coaches	64.6	University sport staff	53.8
Sportmates	45.9	Sport managers	23.8	Academic staff	35.2
Friends	41.3	No one/don’t know	16.3	Professors	35.2
Sisters/brothers	31.9	Sport psychologist	9.6	Administrative staff	28.2
Classmates	21.2	Medical doctor	5.4	No one/don’t know	14.9
No one/don’t know	3.0	Parents	2.3	Parents	0.5
Partner	2.3	University	0.5	Sport federation	0.2
Sponsor	0.5	Career counsellor	0.2		
Agent	0.2	National Olympic Committee	0.2		
Grandparents	0.2				
Workmates	0.2				
City government	0.2				
National Olympic Committee	0.2				

## Discussion

For the first time, the present study attempted to investigate the global dual career phenomenon through the international student-athletes’ point of view by means of an online survey administered to student-athletes who participated in the 2017 Summer Universiade in Taipei (Taiwan). In particular, the main findings deriving from the descriptive and inferential statistical approaches highlighted: 1) a difference between continents for the time spent in sport and university engagement, and in transferring from home to the training venue; 2) a higher amount of time in sport engagement for individual sports student-athletes compared to their team sports counterparts; 3) a difference between university major categories on the time spent in university engagement; 4) and a differences between continents regarding the athletes’ familiarity and awareness of dual career policies, programmes, measures, initiatives and availability of policy documents that facilitate their elite sports and studies paths. Furthermore, the athletes declared that their dual career impacts both on sport- and university-related outcomes despite they receive support mainly from parents, coaches, and academic staff.

In addressing the diversity of dual career across continents, this study represents a valuable starting point for initial tentative speculations on dual career in a globalized elite sports environment and contributes to reveal a wide spectrum of athletes’ experiences and perceptions at micro, meso, and macro dual career levels. To note, the respondents declared a high athletic level and a long elite sport career [45], substantiating that the Universiade is a tremendous opportunity to investigate various dual career issues of the best worldwide student-athletes. As expected, some differences between continents emerged, especially related to the athletes’ engagement in sport and university, in the logistics affecting the time for transferring from home to the training venue, and their awareness of dual career opportunities and rights. In fact, heterogeneous and specific cultural contexts highly influence the regulation of dual career systems, the provision of support programmes, and the dual career culture in general [14,23].

Student-athletes pursuing a dual career path are exposed to a concomitant sport and university load to meet all the requirements of both domains. Furthermore, time necessary to transfer from home and university to the training venue should be considered, since it can affect the daily schedule of student-athletes. On average, a similar amount of time for sport (21±10 hr·week^-1^) and university (22±21 hr·week^-1^) engagement has been registered worldwide. However, differences among continents have been found, reflecting distinct exposures of student-athletes to sport and university loads. In particular, compared to the other continents, African respondents declared the lowest engagement in sport, although showing a similar amount of time for university engagement with respect to their American, European, and Oceanic student-athletes. Conversely, Asian student-athletes showed the lowest amount of time spent at university compared to that reported by their counterparts from all the other continents. Indeed, different commitments to sport and university might also reflect successful outcomes in international sport events. In fact, the 2017 Summer Universiade medal ranking [46] included five Asian countries in the top ten positions. Despite the success in sport performance depends on a variety of factors encompassing the athlete’s technical, tactical, physiological, and psychological/social characteristics [47], it could be speculated that a reduced burden of academic work could facilitate athletes in focusing more on sport, thus avoiding concurrent dual career demands in favor of the immediate gratification of a sport success. Because academic engagement and performance increase in presence of a positive contextual dual career climate and dedicated staff, a thorough understanding dual career in globalized contexts could be obtained through future comparative studies [14,23].

In the European dual career literature, differences in relation to sport typology have been reported on motivation towards dual career [48,49], dual career competencies [28], and university major orientation [50]. The present study adds to this knowledge reporting differences for sport engagement, with student-athletes from individual sports dedicating more time (around 3 hr·week^-1^) to sport with respect to their team sports counterparts. Two speculations could explain these findings: A high training volume needed for excelling in some individual sports (e.g., endurance, swimming, gymnastics), and the possibility to organize training on individual basis. Therefore, sort-specific dual career paths are strongly envisaged.

In the literature [51], the relevant role that sport holds in the athletes’ lives has been considered to undermine the athletes’ choice of the university major. The present study confirms that student-athletes less likely engage in majors related to Physical Sciences and Engineering [51], which also showed the highest amount of time in university engagement. In considering that the university major did not affect the time for sport engagement, it could be speculated that the present findings mirror the general enrolment trends of university students based on individual attitudes and career planning rather than athletes’ choices towards topics considered suitable to be combined to their actual sport career. In fact, despite a degree in Sport Sciences could be considered a facilitator for future sport-related careers [52] and less demanding degree programmes have been reported in athletes [53], the present study revealed a lack of a clear impact of university major on dual career phenomenon. Furthermore, the percentage of graduated student-athletes aiming to pursue master’s and PhD degrees substantiates that this population considers education an important aspect to support a future employment at the end of the sport career [30,33,54]. In particular, the high percentage of bachelor students >23 years could mirror personalized part-time boarding academic paths offered at some universities to allow student-athletes to combine studies and competitions. These findings corroborate the inappropriateness of considering the academic career of elite athletes in general terms and the need of further research on the academic motivation of athletes [33].

The present results confirm and expand those reported by a European study involving experts of 25 Member States that indicated around 40 hours of work dedicated to sport and study [55]. In addition, the extra time required to transfer from and to the training venue makes the time devoted to dual career commitment a relevant aspect for student-athletes trying to manage their daily life. Therefore, it is urgent to raise the awareness of academic institutions and sport bodies to provide a supportive environment, including specific arrangements, flexible academic and sport timetables, online study opportunities, tutoring and welfare services to help student-athletes overcoming the barriers related to time management [3,4] and to acquire life skills [56]. In particular, for American student-athletes, it was clearly evident a higher amount of time to transfer from home to training venue, compared to other continents, possible affecting their daily schedule. Therefore, in considering that student-athletes might tend to privilege sport when organizing their life, the universities should implement the availability of training centers to increase the efficiency of the training-study time schedule, crucial for the success of dual career paths [57].

In line with the literature [56,58–60], the present results confirm a constant tension between the academic, sporting, and social components of the student-athlete’s life, which determines a limited social time and overload feelings. Indeed, to maintain their dual career paths student-athletes have to develop their own approach when sport or academics inevitably have to be prioritized, unavoidably compromising other aspects of their social lives [59,61]. Despite the literature highlights a very limited flexibility around training schedules [7,59], in the present study the student-athletes reported similar time away from sport and academic duties to achieve their successful dual career potentials in presence of conflicting schedules. Thus, the culturally dominant discourse that flexibility should pertain mainly the educational institutions is challenged and the supportive role of quality coaching towards rearranging training schedule in relation to academic duties of their student-athletes are envisaged.

Surprisingly, the analysis of student-athletes’ familiarity and awareness of dual policies and programmes highlighted that student-athletes tend to ignore the legislative framework to ensure dual career of athletes. In particular, negative responses emerged also from student-athletes competing for countries where multiple initiatives are adopted. In fact, even some student-athletes from the United States seem to ignore that the National Collegiate Athletic Association established in 1994 the CHAMPS/Life Skills programme, further implemented in 2016 to provide daily oversight and operation of programming for student-athletes [62]. This holds true also for European student-athletes from countries identified in a recent study on the minimum quality requirements for dual career services [3] as having dual career policy documents in place (e.g., Austria, Belgium, Croatia, Czech Republic, Denmark, Estonia, Finland, Germany, Great Britain, Hungary, Ireland, Latvia, Portugal, and Slovenia) or adopting initiatives at local level (e.g., Italy). Indeed, communication on dual career opportunities, programmes, and polices is crucial to make responsible and autonomous student-athletes in evaluating their strengths, weaknesses, and possible solutions in pursuing a dual career. Actually, the need to resolve this lack of information has been mentioned in previous studies [23,29]. In this respect, policy makers are urged to stimulate the media to communicate the role of the student-athletes and particular attention should be devoted to inform talented and elite athletes on the dual career policies and opportunities available in their country as well as in other ones. This is particularly relevant in light of the relevant phenomenon of migrating athletes who relocate to pursue their sport and/or academic careers.

Despite student-athletes should have the right to attend tertiary education to be safeguarded and helped reaching their full potential in life [16], the respondents’ perceptions substantiate that a high number of their peers does not pursue education at university level and many student-athletes still deserve the same opportunities of those who receive a dual career support [3]. In particular, a need of information, counselling and tutorship at sport and academic levels emerged, deemed crucial to acquire problem-solving skills related to the management of their dual career [63]. Therefore, it is essential to have specific qualifications for competent dual career staff (e.g., academic and sport dual career tutor) to present student-athletes with individualized or group counselling regarding programmes, education, employment, career planning, and to navigate obstacles during their years of competitive sport [3,4,29]. Moreover, the student-athletes’ voices were mainly directed towards a financial support, educational flexibility, and sport facilities close to their university. Actually, very few elite student-athletes are financially independent through their sports earnings, with athletes competing in nonrevenue-generating sports or in sports considered “minor” at national level significantly differing from their revenue-generating sport counterparts [56]. Tangential to the theme related to the transfer from and to the training site considered in the previous section, sports facilities situated close to the educational facilities are essential to optimize the student-athlete’s time management and to enhance cooperation between sport and academic staff. Thus, to have student-athletes equally benefiting of educational bursary and scholarships, living accommodation, specialized dual career staff, and facilities and services at educational and sport levels, a worldwide minimum standard is strongly envisaged [3].

A dual career starts at young ages and spans through the developmental years of the individual, during which training, competition, and school demands significantly vary in typology, volume, intensity, and organization according to the sport and education policy contexts of the different countries. Research has highlighted several career stages and transitions related to the athletic, psychological, psychosocial, academic/vocational, and financial dimensions of the student-athletes, occurring at different times and having a reciprocal influence on the holistic development of the individual [23, 64]. Despite actors playing a role at the most proximal dimension of a student-athlete’s dual career support network, their role might vary throughout the developmental stages towards elite sport. Indeed, sport parenting is well recognized to play a unique and important role within both the sport and academic contexts of their children [65]. In fact, positive emotional, financial and social parental support, and encouragement is deemed crucial to the student-athlete’s motivation and success in combining sport and education, whereas negative parental attitudes might put student-athletes at risk of psychosocial challenges, and sport or academic dropouts [66–68]. In this study, respondents emphasized the prevalent function parents also have with respect to academic education. However, at present parents do not have the opportunity to be empowered in their specific role as dual career supporters and a specific educational programme is strongly envisaged [69]. Besides parents, coaches are considered central to influence the student-athlete inside as outside of sport [70], having a mentoring role in the student-athlete’s life as a dual career supporter [71]. This is particularly important when elite student-athletes enter academic institution abroad [72]. Therefore, sport federations are urged to include dual career classes in the educational and refreshment programmes for coaches and sport staff, who are the expert of the sport-specific requirements and could help student-athletes negotiating their educational duties [73]. Finally, also university sport staff and professors should be aware of the burden placed on student-athletes and be ready to rethink their role as facilitators in the context of an effective lifelong learning perspective. Therefore, specialized staff should be employed as formal dual career tutors at sport and education levels to counsel student-athletes, coaches, and professors regarding individualized student-athletes’ whole-life-development-plans and progresses [3,63].

The present study had some limitations which need to be addressed and could serve as a guidance for future research. Despite a considerable amount of participants at the 2017 Summer Universiade (7377 athletes), the rate of responses was low (5.8%). Possible explanations could be: 1) the request of participation was sent at the end of the event when most of the students-athletes and national delegated were on vacation; 2) student-athletes of countries where sport and education are combined (e.g., mostly North America and Oceania) might have considered the dual career issue not relevant because given for granted; and 3) student-athletes with a limited command of English could have ignored the invitation to participate. Moreover, the unbalanced recruitment of student-athletes regarding continents, sport typology, and university major might limit the generalizability of findings [40].

To strengthen the evidence on the interaction between the sport and academic contexts and their local-global nexus, further studies should consider the stratification of a large sample of student-athletes [40]. Furthermore, future research, actions, programmes, and monitoring activities at sport and educational levels focused on the individual, inter-personal, environmental, and policy dimensions affecting constructive dual career paths are strongly needed. This further effort could strongly contribute to the progression of the process towards the establishment of a culture that supports student-athletes for a relevant role in society at the end of their sport career.

## Conclusions

A highlight of this research was that the online FISU-EAS survey provided a valuable glimpse into the perception of dual career in student-athletes competing for their respective African, American, Asian, European, and Oceanic countries in the international multi-sport Universiade event. Albeit preliminary, these findings are likely to be very meaningful for dual career research domain and could foster further studies based on an international approach. In particular, when international university sport competition is at stake it is assumed that student-athletes have equal opportunity and support to their holistic development and to advance their potential in sport and in society through a higher education [16]. Conversely, relevant cultural, organizational, and/or economical differences in relation to the sport-specific and education-specific dual career programmes, services and support are present due to competence in the field of sport and education mainly remaining with countries [3,10,11,58]. To reinforce the need of a minimum standard for dual career programmes and services, it is necessary that the sport organizations and policy makers incorporate this issue in their political agenda [4]. In this respect, international sport and governmental bodies have the opportunity to recommend formal dual career agreements aiming at establishing well-structured cooperation systems between stakeholders, educational programmes for specialized personnel, and a systematic monitoring of the effectiveness of programmes. Furthermore, to strengthen the potential of the athletes of the future, the stakeholders are strongly recommended to join a dual career network, which supports transnational cooperation and the sharing of knowledge and best practices through extensive communication from policy to practice [20].

In this study, the majority of the student-athletes was not only competing at the highest international level (e.g., World Games, World Cups, and Olympic Games) but also enrolled in a variety of university majors and academic levels, from bachelor to PhD degrees. It is clear that elite student-athletes with comparable amount of experience and time practicing sports have the most meaningful experience of the complexities of dual career paths at tertiary levels. Indeed, the needs of the individual student-athlete and her/his whole-life development plan can be very unique. However, irrespectively of country- and sport typology-related aspects, engagement in both elite sport and university education resulted uniformly highly demanding. Therefore, it is urgent to consider the valuable insights from the student-athletes to avoid that the sport success comes at the expense of the educational attainment. In particular, some major needs emerged: 1) to establish an international agreement on the minimum standard of dual career services; 2) to provide specific educational programmes for dual career service providers able to negotiate flexible requirements at academic and sport levels, as well as for those having a close relationship with the student-athlete and a strong supportive dual career role (e.g., parents, coaches, and university staff); and 3) to inform the student-athletes on their dual career rights, policies, programmes, services, financial resources, and logistic support in place in their home country, in addition to availability of assets and opportunities as transnational student-athletes. In this respect, it is strongly envisaged a strict cooperation between dual career stakeholders and media in developing supporting materials such as pamphlets, brochures, and communication campaigns. The common efforts of FISU and EAS in fostering the cultural process that sustains elite student-athletes in education should be supported by an enhancement of dual career policies at global level.

## Supporting information

S1 Appendix The FISU-EAS questionnaire dual career of athletes (Dichotomous and single or multiple response checklist type are under brackets). (DOCX)

## References

[1] Park S, Lavallee D, Tod D. Athletes' career transition out of sport: A systematic review. Int Rev Sport Exerc Psychol. 2013;6(1): 22–53. 10.1080/1750984X.2012.687053

[2] Torregrosa M, Ramis Y, Pallarés S, Azócar F, Selva C. Olympic athletes back to retirement: A qualitative longitudinal study. Psychol Sport Exerc. 2015; 21: 50–56.

[3] Amsterdam University of Applied Sciences, Birch Consultants, the Talented Athlete Scholarship Scheme, the Vrije Universiteit Brussel, & European Athlete as Student Network. Study on the minimum quality requirements for dual career services. Research Report. 2016. Available from: https://ec.europa.eu/sport/news/2016/study-minimum-quality-requirements-dual-careers-published_en.

[4] Capranica L, Guidotti F. Qualifications/dual careers in sports: Research for Cult Committee of the European Parliament: Directorate-General for internal policies. Policy Department. Structural and cohesion policies: Cultural and Education. 2016 Available from: http://www.europarl.europa.eu/RegData/etudes/STUD/2016/573416/IPOL_STU(2016)573416_EN.pdf.

[5] Australian Government. Australian Institute of Sport, Elite Athlete Friendly University program. 2018. Available from: https://www.ausport.gov.au/ais/personal_excellence/university_network/elite_athlete_friendly_university_program.

[6] Canadian Sport Institute. Education, Career and Transition: Sportsgrad Program. 2018. Available from: http://csicalgary.ca/life-services/education-career-and-transition.

[7] Ryan C, Thorpe H, Pope C. The policy and practice of implementing a student–athlete support network: a case study. International Journal of Sport Policy and Politics. 2017;9: 415–430. 10.1080/19406940.2017.1320301

[8] National Collegiate Athletic Association. Student-Athlete. 2018. Available from: http://www.ncaa.org/student-athletes.

[9] Aquilina D, Henry I. Elite athletes and university education in Europe: a review of policy and practice in higher education in the European Union Member States. Int J Sport Pol. 2010;2(1): 25–47. 10.1080/19406941003634024

[10] Sum RKW, Tsai HH, Ching Ha AS, Cheng CF, Wang FJ, Li M. Social-ecological determinants of elite student athletes’ dual career development in Hong Kong and Taiwan. SAGE Open. 2017;7(2): 2158244017707798. 10.1177/2158244017707798

[11] Tshube T, Feltz DL. The relationship between dual-career and post-sport career transition among elite athletes in South Africa, Botswana, Namibia and Zimbabwe. Psychol Sport Exerc. 2015;21: 109–114. 10.1016/j.psychsport.2015.05.005

[12] Duffy PJ, Lyons DC, Moran AP, Warrington GD, MacManus PC. How we got here: Perceived influences on the development and success of international athletes. Ir J Psychol. 2006;27(3–4): 150–167. 10.1080/03033910.2006.10446238

[13] Li M, Sum RKW. A meta-synthesis of elite athletes’ experiences in dual career development. Asia Pacific Journal of Sport and Social Science. 2017;6(2): 99–117. 10.1080/21640599.2017.1317481

[14] Guidotti F, Cortis C, Capranica L. Dual career of European student-athletes: A systematic literature review. Kinesiol Slov. 2015;21(3): 5–20.

[15] European Commission. White Paper on Sport. (2007). Available from: https://eur-lex.europa.eu/legal-content/EN/TXT/?uri=LEGISSUM%3Al35010.

[16] European Commission. guidelines on dual careers of athletes recommended policy actions in support of dual careers in high-performance sport. 2012. Available from: http://ec.europa.eu/assets/eac/sport/library/documents/dual-career-guidelines-final_en.pdf.

[17] Council of the European Union. Conclusions of the Council and of the Representatives of the Governments of the Member States, Meeting within the Council, on Dual Careers for Athletes. 2013. Available from: https://eur-lex.europa.eu/legal-content/EN/TXT/?uri=celex%3A52013XG0614%2803%29.

[18] European Parliament. Report on an Integrated Approach to Sport Policy: Good Governance, Accessibility and Integrity (2016/2143(INI). 2016. Available from: http://www.europarl.europa.eu/sides/getDoc.do?pubRef=-//EP//TEXT+REPORT+A8-2016-0381+0+DOC+XML+V0//EN.

[19] International Olympic Committee. IOC Athlete Career Programme (ACP). 2005. Available from: https://www.olympic.org/athlete365/wp-content/uploads/2015/11/CIO1444-1_Programme_interactif_vfinale_EN.pdf.

[20] Capranica L, Foerster J, Keldorf O, Leseur V, Vandewalle P, Abelkalns I, et al The European Athlete as Student network ("EAS"): Prioritising dual career of European student-athletes. Kinesiol Slov. 2015;21(2): 5–10.

[21] Caput-Jogunica R, Ćurković S, Gordana B. Comparative analysis: support for student-athletes and the guidelines for the university in southeast Europe. Sport Science. 2012;5: 21–26.

[22] Stambulova NB, Wylleman TV. Dual career development and transitions. Psychol Sport Exerc. 2105;21: 1–134. 10.1016/j.psychsport.2015.05.003

[23] Stambulova NB, Wylleman P. Psychology of athletes' dual careers: A state-of-the-art critical review of the European discourse. Psychol Sport Exerc. 2018;42: 74–88. 10.1016/j.psychsport.2018.11.013

[24] Cabrita TM, Rosado AB, Leite TO, Serpa SO, Sousa PM. The relationship between athletic identity and career decisions in athletes. J Appl Sport Psychol. 2014;26(4): 471–481. 10.1080/10413200.2014.931312

[25] Harrison CK, Tranyowicz L, Bukstein S, McPherson-Botts G, Lawrence SM. I am what I am? The Baller Identity Measurement Scale (BIMS) with a Division I football team in American higher education. Sport Sci Health. 2014;10(1): 53–58. 10.1007/s11332-014-0171-3

[26] Martin BE, Harrison CK, Stone J, Lawrence SM. Athletic voices and academic victories: African American male student-athlete experiences in the Pac-Ten. J Sport Soc Issues. 2010;34(2): 131–153. 10.1177/0193723510366541

[27] De Brandt K, Wylleman P, Torregrossa M, Defruyt S, Van Rossem N. Student-athletes’ perceptions of four dual career competencies. Rev Psicol Deporte 2017;26(4): 28–33. http://www.redalyc.org/articulo.oa?id=235152047006

[28] Graczyk M, Wylleman PI, Nawrocka A, Atroszko P, Moska W, Tomiak T, et al The importance of the type of sport and life experience in the dual career in elite sport based on the analysis of Poland. Balt J Health Phys Act. 2017;9(4): 135–146. 10.29359/BJHPA.09.4.11

[29] de Subijana CL, Barriopedro M, Conde E. Supporting dual career in Spain: Elite athletes' barriers to study. Psychol Sport Exerc. 2015;21: 57–64. 10.1016/j.psychsport.2015.04.012

[30] Fortes PC, Rodrigues G, Tchantchane A. Investigation of academic and athletic motivation on academic performance among university students. Int J Trade Economics Finance. 2010;1(4): 367–372. 10.7763/IJTEF.2010.V1.65

[31] Keegan RJ, Spray CM, Harwood CG, Lavallee DE. A qualitative synthesis of research into social motivational influences across the athletic career span. Qual Res Sport Exerc Health. 2014;6(4): 537–567. 10.1080/2159676X.2013.857710

[32] Kerstajn R, Lupo C, Capranica L, Doupona-Topic M. Motivation towards sports and academics careers in elite winter sport Slovenian and Italian athletes: The role of internal and external factors. Ido Mov Culture J Martial Arts Anthrop. 2018;18(2): 29–37. 10.14589/ido.18.2.4

[33] Lupo C, Guidotti F, Goncalves CE, Moreira L, Doupona Topic M, Bellardini H, et al Motivation towards dual career of European student-athletes. Eur J Sport Sci. 2015;15(2): 151–160. 10.1080/17461391.2014.940557 25145585

[34] Parker PC, Perry RP, Hamm JM, Chipperfield JG, Hladkyj S. Enhancing the academic success of competitive student athletes using a motivation treatment intervention (Attributional Retraining). Psychol Sport Exerc. 2016;26: 113–122. 10.1016/j.psychsport.2016.06.008

[35] Aunola K, Selänne A, Selänne H, Ryba TV. The role of adolescent athletes' task value patterns in their educational and athletic career aspirations. Learn Individ Differ. 2018;63: 34–43. 10.1016/j.lindif.2018.03.004

[36] Stambulova NB, Ryba TV. Athletes' Careers Across Cultures. London: Routledge; 2013.

[37] Kuettel A, Boyle E, Schmid J. Factors contributing to the quality of the transition out of elite sports in Swiss, Danish, and Polish athletes. Psychol Sport Exerc 2017;29: 27–39. 10.1016/j.psychsport.2016.11.008

[38] Franck A, Stambulova NB. The junior to senior transition: a narrative analysis of the pathways of two Swedish athletes. Research in Sport, Exercise and Health 2019;11(3): 284–98. 10.1080/2159676X.2018.1479979

[39] Rebustini F, Balbinotti MAA, Ferretti-Rebustini REDL, Machado AA. Sport psychometry, participants and invariance: A critical review. J Phys Educ. 2016; 27: e2760 10.4025/jphyseduc.v27i1.2760

[40] Callegaro M, Manfreda KL, Vehovar V. Web Survey Methodology. ‎Thousand Oaks: Sage; 2105.

[41] Deutskens E, De Ruyter K, Wetzels M, Oosterveld P. Response rate and response quality of internet-based surveys: An experimental study. Mark Lett. 2004;15(1): 21–36. 10.1023/B:MARK.0000021968.86465.00

[42] Hooley T, Marriott J, Wellens J. What is online research?: Using the internet for social science research London: Bloomsbury Academic; 2012.

[43] European Research Council Panel Structure. 2019. Available from: https://erc.europa.eu/content/erc-panel-structure-2019

[44] Cohen J. Statistical power analysis for the behavioral sciences London: Routledge; 2013.

[45] Alfermann D, Stambulova N. Career transitions and career termination In Tenenbaum G, Eklund RC, editors. Handbook of sport psychology. Hoboken: John Wiley & Sons Inc; 2007 pp. 712–733.

[46] 29th Summer Universiade Taipei 2017. Available from: https://2017.taipei/home

[47] Bangsbo J. Performance in sports–With specific emphasis on the effect of intensified training. Scand J Med Sci Sports. 2015;25: 88–99. 10.1111/sms.12605 26589122

[48] de Subijana CL, Barriopedro MI, Sanz I. Dual career motivation and athletic identity on elite athletes. Rev Psicol Deporte. 2015;24(1): 55–57. http://www.redalyc.org/articulo.oa?id=235143644012

[49] Fuchs P, Wagner H, Hannola H, Niemisalo N, Pehme A, Puhke R, et al European student-athletes' perceptions on dual career outcomes and services. Kinesiol Slov. 2016;22(2): 31–48.

[50] Tekavc J, Wylleman P, Erpič SC. Perceptions of dual career development among elite level swimmers and basketball players. Psychol Sport Exerc. 2015;21: 27–41. 10.1016/j.psychsport.2015.03.002

[51] Foster SJ, Huml MR. The relationship between athletic identity and academic major chosen by student-athletes. Int J Exerc Sci. 2017;10(6): 915–925. 29170694 10.70252/FIJG1609 PMC5685074

[52] Guidotti F, Capranica L. Le motivazioni verso sport, istruzione e carriera sportiva degli studenti-atleti italiani In Pioletti AM, Porro N editors. Lo sport degli Europei. Milan: Edizioni Franco Angeli; 2013 pp. 104–120.

[53] Fountain JJ, Finley PS. Academic clustering: A longitudinal analysis of a Division I football program. Journal of Issues in Intercollegiate Athletics. 2011;4: 24–41.

[54] Gaston-Gayles JL. Examining academic and athletic motivation among student athletes at a Division I university. J Coll Stud Dev. 2004;45(1): 75–83. 10.1353/csd.2004.0005

[55] PMP Consultants and Institute of Sport and Leisure Policy Loughborough University. Education of young sportspersons (Lot 1) Final Report. Research for the European Commission Dg Education & Culture. Brussels: European Commission; 2004.

[56] Paule AL, Gilson TA. Current collegiate experiences of big-time, non-revenue, NCAA athletes. Journal of Intercollegiate Sport. 2010;3(2): 333–347. 10.1123/jis.3.2.333

[57] Stambulova NB, Engström C, Franck A, Linnér L, Lindahl K. Searching for an optimal balance: Dual career experiences of Swedish adolescent athletes. Psychol Sport Exerc. 2015;21: 4–14. 10.1016/j.psychsport.2014.08.009

[58] Aquilina D. A Study of the relationship between elite athletes' educational development and sporting performance. Int J Hist Sport. 2013;30(4): 374–392. 10.1080/09523367.2013.765723

[59] Cosh S, Tully PJ. Stressors, coping, and support mechanisms for student athletes combining elite sport and tertiary education: Implications for practice. Sport Psychol. 2015;29(2): 120–133. 10.1123/tsp.2014-0102

[60] Ryan C. Factors impacting carded athlete's readiness for dual careers. Psychol Sport Exerc. 2015;21: 91–97. 10.1016/j.psychsport.2015.04.008

[61] Gomez J, Bradley J, Conway P. The challenges of a high-performance student athlete. Irish Ed Studies. 2018;37(3): 329–349. 10.1080/03323315.2018.1484299

[62] National Collegiate Athletic Association. Life Skills. 2018. Available from: http://www.ncaa.org/about/resources/leadership-development/life-skills

[63] Sánchez Pato A, Isidori E, Calderón A, Brunton J. Handbook: An innovative European sports tutorship model of the dual career of student-athletes. UCAM Publ; 2107.

[64] Wylleman P, Reints A. A lifespan perspective on the career of talented and elite athletes: Perspectives on high‐intensity sports. Scand J Med Sci Sports. 2010;20(s2): 88–94. 10.1111/j.1600-0838.2010.01194.x 20840566

[65] Knight CJ, Harwood CG. The role of the entourage in supporting elite athlete performance and educational outcomes. IOC Olympic Studies Centre 2015.

[66] Tamminen KA, Holt NL, Crocker PR. Adolescent athletes: psychosocial challenges and clinical concerns. Curr Opin Psychiatry. 2012;25(4): 293–300. 10.1097/YCO.0b013e3283541248 22569310

[67] Wuerth S, Lee MJ, Alfermann D. Parental involvement and athletes’ career in youth sport. Psychol Sport Exerc. 2004;5(1): 21–33. 10.1016/S1469-0292(02)00047-X

[68] Wylleman P, De Knop P, Ewing M, Cumming S. Transitions in youth sport: A developmental perspective on parental involvement In Lavallee D, Wylleman P, editors. Career transitions in sport: International perspectives Morgantown, WV: Fitness Information Technology; 2000 pp. 143–160.

[69] Knight CJ, Berrow SR, Harwood CG. Parenting in sport. Curr Opin Psychol. 2017;16: 93–97. 10.1016/j.copsyc.2017.03.011 28813364

[70] Mageau GA, Vallerand RJ. The coach–athlete relationship: A motivational model. J Sports Sci. 2003; 21(11): 883–904. 10.1080/0264041031000140374 14626368

[71] Debois N, Ledon A, Wylleman P. A lifespan perspective on the dual career of elite male athletes. Psychol Sport Exerc. 2015;21: 15–26. 10.1016/j.psychsport.2014.07.011

[72] Baghurst T, Fiaud V, Tapps T, Bounds E, LaGasse A. Considerations when coaching the international athlete. International Journal of Kinesiology in Higher Education. 2018;2(3): 76–86. 10.1080/24711616.2018.1425936

[73] Hakkers S. How can sport clubs support a talent’s dual career? Guidebook of best practices in dual career. Final report of the EC-funded project Innovative Clubs for Dual Career (IC4DC) 2019 Available from: https://www.icdc.eu/en/content/documentation

