# Supplementary material for: Dual-career through the elite university student-athletes’ lenses: The international FISU-EAS survey
Source: PLoS One. 2019 Oct 2;14(10):e0223278. doi: 10.1371/journal.pone.0223278 (PMC6774511; doi:10.1371/journal.pone.0223278)
Supplement: S1 Appendix — (DOCX) [file pone.0223278.s001.docx]

S1 Appendix

**The FISU-EAS questionnaire Dual Career of Athletes (Dichotomous and single or multiple response checklist type are under brackets)**

1. I participate in the 2017 Summer Universiade in the sport (single response checklist type)
2. I participate in the 2017 Summer Universiade for the Country (single response checklist type)
3. I am: (dichotomous: female, male)
4. Year of birth (single response)
5. I am enrolled at university level (single response checklist type: Bachelor, Master’s, PhD, Other)
6. My major is (single response checklist type: Art-Art history-Music-Dance-Theatre, Biology-Biochemistry, Business-Economics-Administration, Communication-Multimedia-Journalism, Computer Sciences, Earth-Ocean-Atmospheric-Environmental Sciences, Education, Engineering-Bioengineering, Health, Sciences-Nutrition, History, Language-Foreign languages, Law, Liberal Arts, Literature, Mathematics, Medicine, Military Science, Naval Sciences, Pharmacy, Philosophy, Physics, Political-International Sciences, Psychology, Sociology-Social Sciences, Sport Sciences/Physical education, Statistics, Women-Gender-Sexuality Studies, Other)
7. In which year did you start to compete in competitions of international level? (single response)
8. In which other international competition have you already competed? (multiple response checklist type: Olympic Games, World Championships, World Cups, World University Championships, Other international competitions)
9. How many hours per week are you on average actively engaged in elite sports (training competition, physiotherapy etc.) in the most intense part of the season? (single response)
10. Enter the number of minutes needed to transfer from your home to your training site each way (single response)
11. How many hours per week are you on average actively engaged in university studies during the semester? Enter the number of hours per week (single response)
12. Enter the number of minutes needed to transfer from your university to your training site each way (single response)
13. Which are the problems you are faced in the combination of elite sport and studies? Multiple answers are allowed (multiple response checklist type: Long absence from university classes due to competitions/training camps, Missing single university classes due to training sessions, Missing university tests and exams, Extension of the study time, Reduced training sessions due to university education, Financial uncertainty, Overload to double burden, Little leisure time, Other)
14. Are you familiar with policies, programmes or measures that facilitate the combination of elite sports and studies? (dichotomous: Yes, No)
15. Is there a policy or initiative on ‘Dual Career’ in your country? (single response checklist type: Yes, I know one policy or programme, Yes, there are more than one, None, Don’t know)
16. Are the policy documents in the field of ‘Dual Career’ available in your country? (single response checklist type: Yes, No, Don’t know)
17. Where can one find these policy documents? Multiple answers possible (multiple response checklist type: Internet, Other, Don’t know)
18. What is the scope of 'Dual Career' policy in your country? (single response checklist type: National, Regional, Local, Sports specific, University specific)
19. Which domain is responsible for any ‘Dual Career’ policy in your country? (multiple response checklist type: Sports Institution, Educational Institution, Private company, Government departments, A specific Dual Career organisation, Other)
20. Which organisations regulate the combination of elite sports and studies? (multiple response checklist type: Governmental departments, Universities/schools, National Olympic Committee, Sport Federations, National University Sport Federation, Dual Career organisation, Labour Market, Other)
21. Which level of public authorities is active in any 'Dual Career' policy (multiple response checklist type: National, Regional, Local, Don’t know, Other)
22. How is the success of the 'Dual Career' initiatives evaluated? (multiple response checklist type: It is not evaluated, On the basis of sporting achievements, On the basis of academic achievements, On the basis of sporting and academic achievements, Don’t know, Other)
23. What type(s) of support is available to elite athletes who combine sport with study in your country? (multiple response checklist type: Financial, Sport facilities at/close to the university, Educational flexibility, Tutor at the university, Tutor at the sport organization, Other)
24. In which field do you see possible improvement in the future? multiple response checklist type: Financial, Sport facilities close to the university, Educational flexibility, Tutor at the university, Tutor at the sport organization, Legal/regulations aspects, Communication on existing initiatives, Other)
25. According to your perception, how many elite athletes combine sports and studies in your country? (single response checklist type: 81-100%, 61-80%, 41-60%, 21-40%, <20%, Don’t know)
26. According to your perception, how many elite athletes use the 'Dual Career' support in your country? (single response checklist type: 81-100%, 61-80%, 41-60%, 21-40%, <20%, Don’t know)
27. As an elite athlete, at sport level do you have support for (multiple response checklist type: Sleeping facilities, Restaurant serving healthy-fresh and well-prepared food, Sport facilities of the highest international training level, Physiotherapy, Coach/Strength/Conditioning/Recovery, Nutritionist, Sport Psychologist, Medical support, Tutor for dual career, Career counselling, Rooms to study- ICT and Internet equipped- and to relax, Other)
28. As an elite athlete, at educational level do you have support for (multiple response checklist type: Sleeping facilities, Restaurant serving healthy-fresh and well-prepared food, Sport facilities, Individualized study plan, Flexible class attendance, Flexible exam sessions, Tutor for dual career, Career counselling, Encouraged employability of the individual linked to industry-recognized providers, Rooms to study- ICT and Internet equipped- and to relax, Long-distance learning, Other)
29. At sport level, who provides support for your 'Dual Career' (multiple response checklist type: Coach, Sport managers, Medical doctor, Sport Psychologist, Other)
30. At university level, who provides support for your 'Dual Career' (multiple response checklist type: Professors/Academic staff, Administrative staff, University sport staff, Other)
31. At personal level, who provides support for your 'Dual Career' (multiple response checklist type: Parents, Sister/brother, Friends, Sport teammates, Classmates, Other)
